# Alterations in cortical thickness development in preterm-born individuals: Implications for high-order cognitive functions

**DOI:** 10.1016/j.neuroimage.2015.04.015

**Published:** 2015-07-15

**Authors:** Kie Woo Nam, Nazareth Castellanos, Andrew Simmons, Seán Froudist-Walsh, Matthew P. Allin, Muriel Walshe, Robin M. Murray, Alan Evans, J-Sebastian Muehlboeck, Chiara Nosarti

**Affiliations:** a Department of Psychosis Studies, Institute of Psychiatry, Psychology & Neuroscience, King's Health Partners, King's College London, London, UK; b Department of Neuroimaging, Institute of Psychiatry, Psychology & Neuroscience, King's Health Partners, King's College London, London, UK; c NIHR Biomedical Research Centre for Mental Health at South London and Maudsley NHS Foundation Trust and Institute of Psychiatry, Psychology & Neuroscience, King's Health Partners, King's College London, London, UK; d Montreal Neurological Institute, McGill University, Montreal, Canada; e Centre for the Developing Brain, Division of Imaging Sciences and Biomedical Engineering, King's College London, King's Health Partners, St. Thomas' Hospital, London, UK

**Keywords:** Cortical thickness, MRI, Neuropsychological outcome, Preterm, Support vector machine

## Abstract

Very preterm birth (gestational age < 33 weeks) is associated with alterations in cortical thickness and with neuropsychological/behavioural impairments. Here we studied cortical thickness in very preterm born individuals and controls in mid-adolescence (mean age 15 years) and beginning of adulthood (mean age 20 years), as well as longitudinal changes between the two time points. Using univariate approaches, we showed both increases and decreases in cortical thickness in very preterm born individuals compared to controls. Specifically (1) very preterm born adolescents displayed extensive areas of greater cortical thickness, especially in occipitotemporal and prefrontal cortices, differences which decreased substantially by early adulthood; (2) at both time points, very preterm-born participants showed smaller cortical thickness, especially in parahippocampal and insular regions. We then employed a multivariate approach (support vector machine) to study spatially discriminating features between the two groups, which achieved a mean accuracy of 86.5%. The spatially distributed regions in which cortical thickness best discriminated between the groups (top 5%) included temporal, occipitotemporal, parietal and prefrontal cortices. Within these spatially distributed regions (top 1%), longitudinal changes in cortical thickness in left temporal pole, right occipitotemporal gyrus and left superior parietal lobe were significantly associated with scores on language-based tests of executive function. These results describe alterations in cortical thickness development in preterm-born individuals in their second decade of life, with implications for high-order cognitive processing.

## Highlights

• Individuals born very preterm showed long-term alterations in cortical thickness
• Such alterations affected predominantly frontal and temporal cortices
• Multivariate analysis revealed between-group spatially discriminating features, with 86.5% accuracy
• Longitudinal cortical thickness changes were associated with executive function scores

## Introduction

Developmental patterns of cortical maturation following very preterm birth (VPT, < 32 weeks of gestation) have not been systematically investigated. Results of cross-sectional studies demonstrated alterations in cortical thickness in VPT samples from childhood to adulthood, with the majority of studies conducted during adolescence. Overall, compared to controls, VPT individuals tend to show developmental delay of cortical thinning in parietal, temporal, and frontal cortices (Martinussen et al., 2005; Frye et al., 2010; Nagy et al., 2011; Skranes et al., 2012; Bjuland et al., 2013; Murner-Lavanchy et al., 2014). These cortical alterations are spatially located in brain areas displaying typical patterns of cortical thinning in healthy controls from early childhood to adolescence, which vary in their maturational trajectories according to layers and cortical regions (Shaw et al., 2008).

Cortical thickness, defined as the distance, at a given point, between the inner and outer boundaries of the cortex (MacDonald et al., 2000), is used as a proxy for neuronal density, although cortical thickness in brain areas with different cytoarchitectonical properties is differentially associated with its underlying neuronal structures (la Fougere et al., 2011). The cellular basis for reduction in cortical thickness from childhood to adolescence is not fully understood, but a possible explanation is greater organization of the brain through synaptic pruning, reflecting the refinement of neural circuits involved in cognitive processing and regional specialization of function (Knudsen, 2004; Raznahan et al., 2011). Cortical thickness is therefore used as an index of neurodevelopment (Shaw et al., 2008), and has been associated with cognitive functions (Sowell et al., 2004).

Being sensitive to both genetic and environmental influences (Lenroot and Giedd, 2008), deviations from typical cortical development have been observed in developmental and psychiatric disorders including autism (Ecker et al., 2013) and schizophrenia (Greenstein et al., 2006), but also in individuals experiencing subclinical symptoms including autistic and antisocial traits (Wallace et al., 2012). In VPT samples cortical alterations have been associated with IQ, performance on tasks involving executive function, working memory, perceptual skills, and with internalizing and externalizing behaviour (Martinussen et al., 2005; Lohaugen et al., 2009; Skranes et al., 2012; Zubiaurre-Elorza et al., 2012; Bjuland et al., 2013).

The studies conducted to date have been cross sectional in design, thus excluding mapping of cortical thickness development with age, which enables the investigation of changes within individuals. An increased understanding of whether cortical trajectories can be used to predict neurodevelopmental outcomes represents a challenge with important clinical relevance.

As cortical development continues beyond adolescence (Gogtay et al., 2004; Kochunov et al., 2011; Lebel and Beaulieu, 2011; Petanjek et al., 2011) we studied longitudinal changes in cortical thickness in preterm born individuals and controls, during the time spanning from mid-adolescence to the beginning of adulthood in order to identify within- and between-group developmental patterns. We then identified spatially discriminating features between the groups at early adulthood based on spatially distributed differences in cortical thickness at mid-adolescence, using a pattern classification approach. Finally we explored the association between cortical thickness changes within the spatially distributed regions in which cortical measurements best discriminated between the groups and cognitive outcome at early adulthood.

Based on results of the studies mentioned earlier (Martinussen et al., 2005; Frye et al., 2010; Nagy et al., 2011; Skranes et al., 2012; Bjuland et al., 2013), we hypothesized that preterm-born individuals would display differential longitudinal cortical thickness changes from controls during the late part of adolescence, especially in parietal, temporal and frontal cortices. We further hypothesized that cortical thickness changes in regions vulnerable to long-term alterations following VPT birth would have implications for cognitive outcome, with the potential to represent a biomarker of important changes in cortical development, potentially influencing brain functioning and cognitive outcomes.

## Materials and methods

### Study population

We studied two cohorts of participants born before 33 weeks of gestation and admitted consecutively to the Neonatal Unit of University College London Hospital (UCLH) (Nosarti et al., 2008). The first cohort drew on all individuals born in 1979–82 who were enrolled for long-term follow-up (Nosarti et al., 2004). The second cohort included a selected group of individuals born in 1983–84 (Allin et al., 2007). This selection was necessitated by an expansion in capacity of UCLH in 1983, which prevented inclusion of the entire consecutive series due to limited research resources. The selection criteria were: all individuals born at 28 or less weeks of gestation, as well as a random sample of one in four of those born from 29 to 33 weeks of gestation. A hundred and sixty VPT participants were studied at a mean age of 15 years (i.e., mid-adolescence) and 67 at a mean age 20 years (i.e., early adulthood, 51 of whom were also studied at mid-adolescence). Eighty-eight controls were studied at mid-adolescence and 42 at early adulthood (21 of whom were also studied at mid-adolescence). Inclusion criteria were full-term birth (38–42 weeks) and birth weight > 2500 grams. Exclusions criteria were a history of neurological conditions including meningitis, head injury and cerebral infections.

### Neuropsychological assessment

All study participants were assessed at Time 2 with the following well-validated measures: 1) the Wechsler Abbreviated Scale of Intelligence (WASI) was used to provide estimates of full-scale IQ (Wechsler, 1999); 2) the Visual Reproduction test of the Wechsler Memory Scale-Revised (WMS-R) assessed memory functions, i.e. immediate and delayed recall of non-verbal material (Wechsler, 1987); the California Verbal Learning Test (CVLT) examined verbal memory, and specifically short term memory (Recall, list A), interference of prior learning on new learning and memory (Recall, list B) and recognition (Recognition hits) (Dellis et al., 1987); (3) executive function (EF) was assessed with two language based tests: the Controlled Oral Word Association Test (COWAT) (Benton and Hamsher, 1976), which measures phonemic fluency, mental flexibility and the ability to use different cognitive strategies, such as clustering (Spreen and Strauss, 1991); and the Hayling Sentence Completion Test (HSCT), which measures response initiation and inhibition (Burgess and Shallice, 1997). ‘Global EF’ and ‘Global memory’ scores were then calculated as the sum of domain-specific Z scores; for VPT participants these were obtained using means and SDs from controls, which by default were set to 0 and 1. Only variables where VPT individuals showed significant differences from controls were used, therefore ‘Global EF’ was made up of HSCT (Scaled) and COWAT scores and ‘Global memory’ was made up of CVLT (Recognition hits) and WMS (Immediate and Delayed) scores.

### Magnetic resonance imaging

At mid-adolescence assessment, MRI was performed at two sites. For the 1979–82 cohort and controls a 1.5 Tesla GE Signa Horizon machine (General Electric Medical Systems, Milwaukee, WI, USA) was used at the Institute of Neurology, London. The 1983–84 cohort and controls were scanned using a 1.5 Tesla GE Signa N/Vi system at the Maudsley Hospital, London. At both sites, three-dimensional T1-weighted MR images were acquired in coronal plane, with the spoiled gradient recalled pulse sequence (flip angle 35°, field of view 240 mm, echo time 5 ms, repetition time 35 ms). Each image contained 124 slices with a matrix size of 256 × 256, slice thickness of 1.5 mm and slice gap of 0 mm.

At early adulthood assessment, all participants were scanned at the Maudsley Hospital with the same scanning protocol used at mid-adolescence.

Quality control was carried out using previously described criteria to ensure adequate quality of the T1-weighted volume images, such as avoidance of wraparound artefacts and minimal levels of subject motion (Simmons et al., 2011).

### Cortical surface extraction

The cortical surfaces were extracted from the T1-weighted MR images using the CIVET pipeline (version 1.1.9) (Ad-Dab'bagh et al., 2006; Zijdenbos et al., 2002), according to the following steps: 1) T1-weighted MR images were linearly registered to MNI-Talairach stereotaxic space using the ICBM152 volumetric template as the registration target (Collins et al., 1994). 2) Images were corrected for signal intensity non-uniformity (Sled et al., 1998) and 3) a brain mask was calculated from each input image (Smith, 2002). 4) Then, images were segmented into grey matter, white matter, cerebrospinal fluid (CSF) and background (Zijdenbos et al., 1998) and 5) partial volumes in each voxel was estimated (Tohka et al., 2004). 6) A *non-linear* transformation was calculated from the images in stereotaxic space to the ICBM152 template, and major structures were identified (Collins et al., 1995). 7) Cortices were extracted using a fully automated method, known as Constrained Laplacian Anatomic Segmentation using Proximity (CLASP) algorithm. Cortical extraction was performed in three steps in stereotaxic space aligned with the ICBM152 template (Kim et al., 2005). First, white matter surface was established by deforming a spherical polygon model to the border between grey and white matter. Then, a Laplacian field was formed between the white matter surface and the inner boundary of the CSF. Lastly, grey matter surface was initiated by forming itself on the white matter surface and expanded, guided by the Laplacian field serving as a path for expansion, until reaching the inner boundary of the CSF. Each surface (i.e. grey or white matter) was a triangular mesh composed of 81,924 vertices (i.e. cortical points) with the first half being in the left hemisphere and second half in the right. 8) The cortical surfaces were transformed back into native space, in which cortical thickness was calculated. Each vertex on the grey surface was linked with a corresponding vertex on the white surface, and the distance between each pair was defined as cortical thickness at a given vertex (Kim et al., 2005). 9) Cortical thickness values were smoothed using a 20 mm full width at half maximum kernel (Chung and Taylor, 2004). 10) Finally, the cortical surfaces of all brains were non-linearly registered to the ICBM152 surface template. This achieved correspondence of vertices across subjects (Robbins, 2003).

### Statistical analyses

We first compared cortical thickness at mid-adolescence assessment and at early adulthood assessment between VPT individuals and controls. Within group longitudinal changes in cortical thickness between the two time points were also explored. Subsequently, multivariate pattern classification methods were used to study spatially discriminating features between the two groups at early adulthood based on spatially distributed differences in cortical thickness at mid-adolescence. In order to investigate the functional significance of cortical thickness maturational processes, cortical thickness changes between the two time points in those areas highlighted by multivariate pattern classification analysis were correlated with cognitive scores at early adulthood.

#### Cross-sectional and longitudinal analyses (univariate measure)

All available brain images were analysed using the SurfStat toolbox (http://www.stat.uchicago.edu/faculty/InMemoriam/worsley/research/surfstat/) under MATLAB (version R2012b). Univariate general linear models were fit with Y as a dependent variable which represented cortical thickness at each vertex. Fixed effects models were fit for the cross-sectional analysis (Y = β_1_X_1_ + β_2_X_2_ + β_3_X_3_ + c, where X_1_ represents the participant's age at scan, X_2_ is a nominal variable, indicating whether or not each participant was born very preterm, and X_3_ represents the sex of the participant, the β terms are unknown parameter estimates for the corresponding X terms and c is a constant term). Mixed effects models were fit for the within-group longitudinal analysis (Y = γZ + β_1_X_1_ + β_2_X_2_ + c, where Z is a design matrix representing the random effect of each subject, X_1_ represents the time point at which each subject was scanned (15 or 20 years), X_2_ represents the sex of the participant, γ is an unknown vector of parameter estimates for the random variable Z, the β terms are unknown parameter estimates for the corresponding fixed variables X and c is a constant term). Finally, Time point-by-group interaction was modelled (Y = γZ + β_1_X_1_ + β_2_X_2_ + β_3_X_3_ + β_4_ (X_1_ * X_2_) + c. where Z is a design matrix representing the random effect of each subject, X_1_ represents the time point (15 or 20 years), X_2_ is a nominal variable, indicating group (control or preterm), X_3_ represents the sex of the participant, γ is an unknown vector of parameter estimates for the random variable Z, the β_1,_ β_2_ and β_3_ terms are unknown parameter estimates for the corresponding fixed variables X_1_, X_2_ and X_3_, β_4_ is the unknown parameter estimate for the interaction term (X_1_ * X_2_) and c is a constant term).

In all analyses, white noise and participant's sex were used as nuisance variables. In cross-sectional analyses only, participant's age at scan was also used as a nuisance variable. For longitudinal analyses only, subject was also included as a random-effect variable (variable γZ), in order to account for within-subject correlation inherent in repeated measures (Bland and Altman, 1995).

**Fig. 1:**
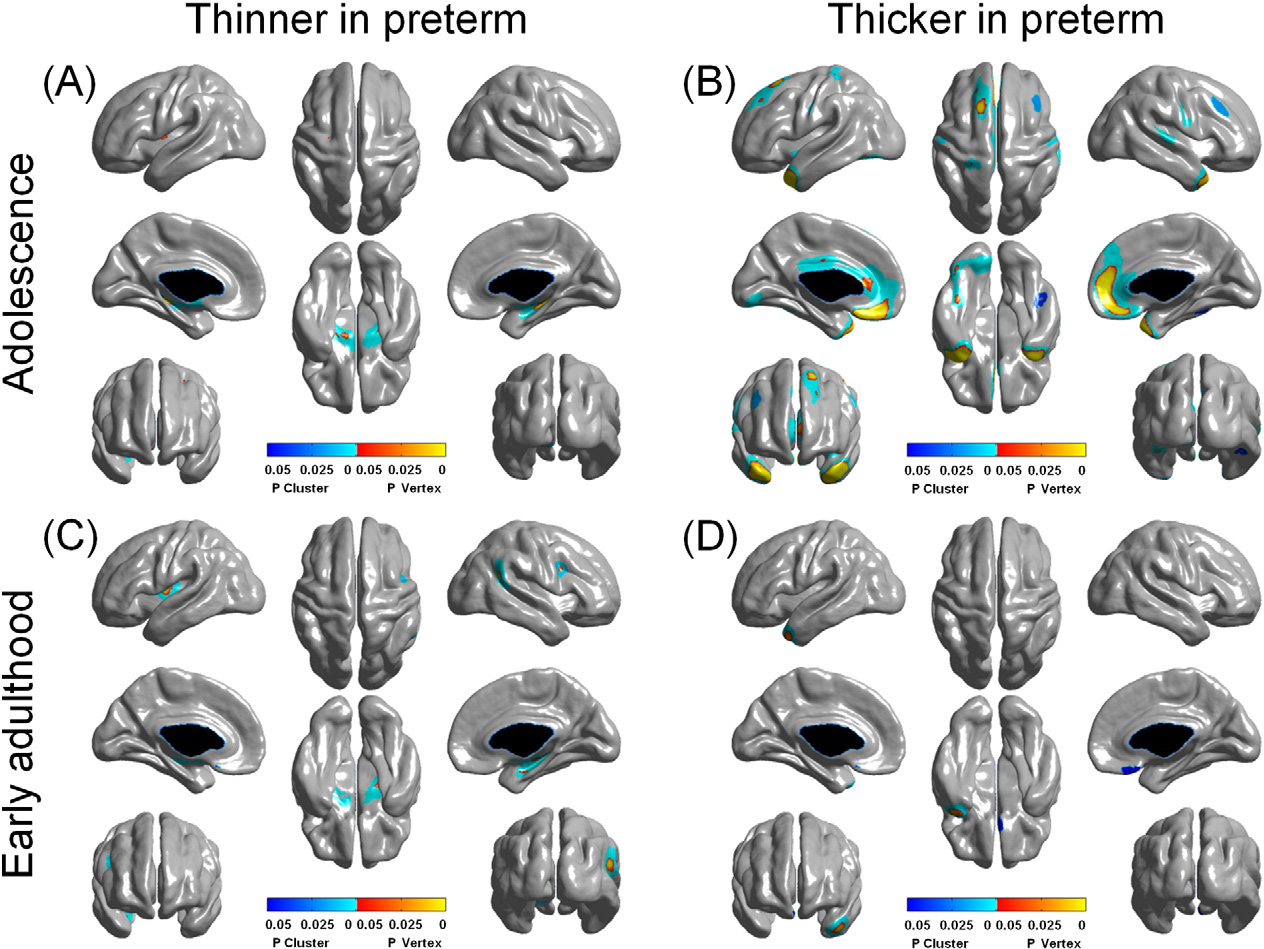
Mean differences in cortical thickness between preterm-born individuals and controls at mid-adolescence and early adulthood.

**Fig. 2:**
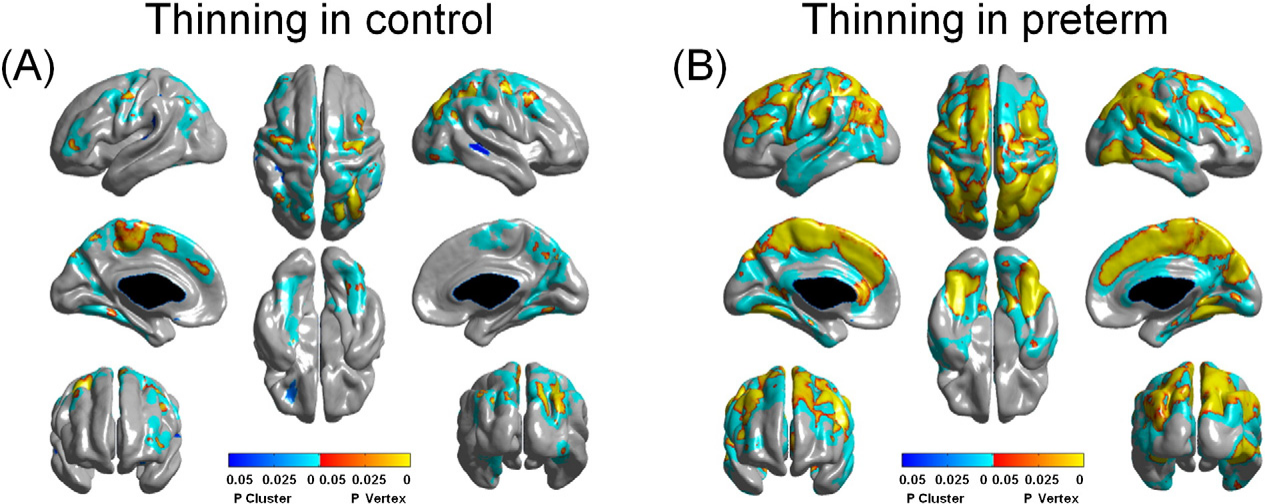
Regions where cortical thickness significantly decreased from mid-adolescence to early adulthood in control (a) and VPT (b) groups.

A one-tailed vertex-wise *T* test was carried out for each contrast within each variable of interest. This produced a T statistic value for each vertex. These were corrected for multiple comparisons based on random field theory (Worsley et al., 1999), which took into account the regional smoothness across neighbouring vertices. Clusters and vertices showing statistically significant differences were identified in different ways. Clusters were first defined with random field theory, and a subset of clusters was identified that had corrected cluster p < 0.05, calculated using Cao (1999)'s method. Vertex threshold (corrected vertex p < 0.05) was calculated with random field theory and Bonferroni corrections, and the smaller (i.e., more lenient threshold) of the two was chosen. These significant vertices were termed “cluster peaks” in Figs. 1 and 2 because they represented the top vertices in a cluster.

For the cross-sectional analysis only, effect sizes represented as Cohen's d (= {Preterm mean − Control mean} / SD) were calculated for each area where significant group differences were observed.

A previous study with the same dataset did not detect a significant effect of MRI acquisition site on data analyses (Nosarti et al., 2008), therefore scan site was not modelled in the current analyses.

#### Longitudinal analysis (multivariate measure)

A multivariate measure, support vector machine (SVM) (Vapnik, 1995), was used to study spatially discriminating features between the two groups (preterm individuals and controls) at early adulthood based on spatially distributed differences in cortical thickness at mid-adolescence. Multivariate measures are advantageous compared to a univariate measures for a number of reasons. Firstly, they may be sensitive to different portions of variance within data; Secondly, multivariate techniques may be more powerful than univariate approaches, as they can reduce noise from single vertices by integrating information from multiple noisy vertices (Davis et al., 2014).

**Fig. 3:**
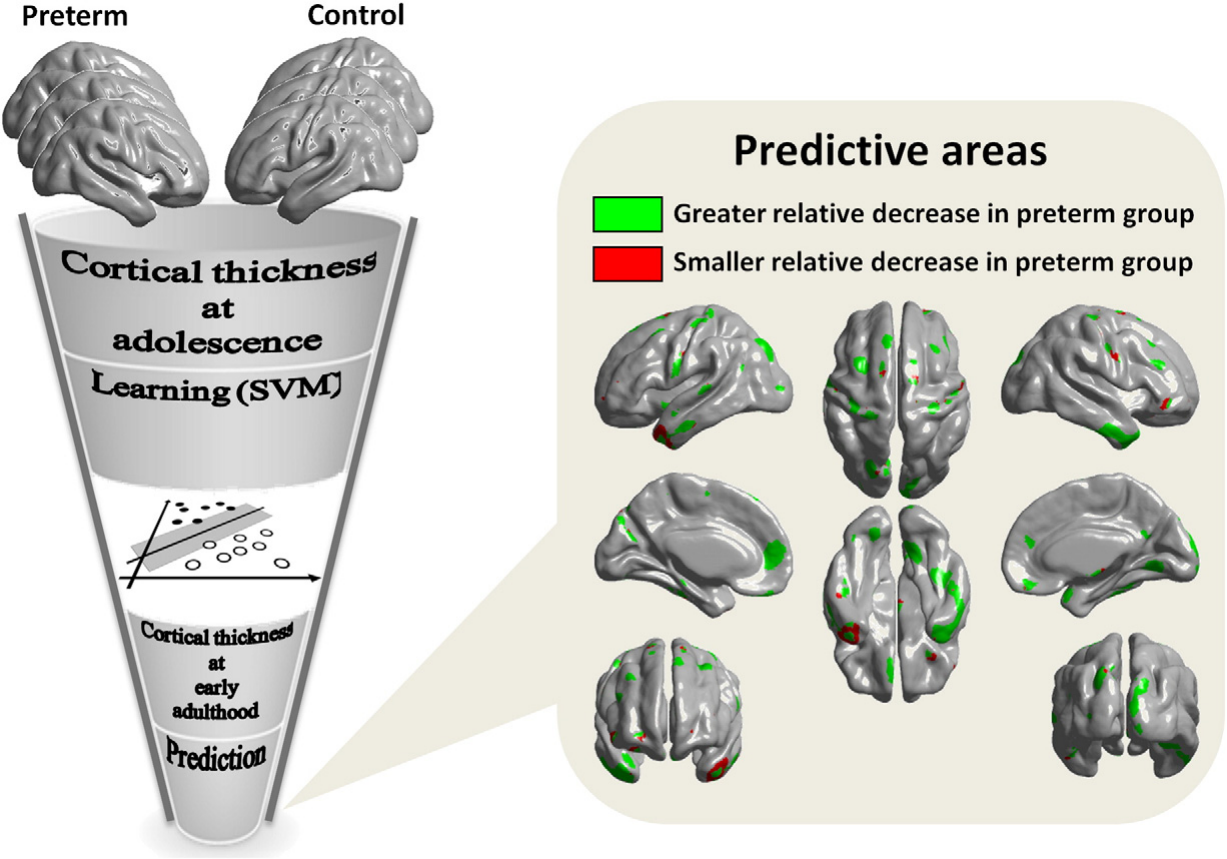
Prediction of regional cortical thickness alterations at early adulthood based on SVM weight vectors acquired from group classification at mid-adolescence.

Given the longitudinal nature of this work, and the implications of suboptimal development following VPT birth, we aimed to investigate specific cortical thickness alterations that conferred a high risk of functional impairments in adulthood using multivariate methods, which would allow us to make inferences at the individual, rather than the group level. For SVM analysis, only images for participants who were scanned at both time points were used. Analysis comprised two phases: i) the training phase, where a classifier was trained using group-labelled data, cortical thickness at mid-adolescence in 21 controls and 51 preterm individuals; ii) the testing phase, where unseen data, i.e., cortical thickness at early adulthood in the same subjects used in the training phase, were introduced to be classified. A linear kernel was used to represent the data, reduce computational cost and improve classification accuracy (i.e., overall rate of correct classification). A recursive feature test was applied to ensure discrimination accuracy was not due to overfitting. In order to test the original classification accuracy and to test if results were independent of the original training data, we carried out a leave-one-out cross validation loop (Geisser, 1993). The goal of using our longitudinal data set for both phases of classification (cortical thickness at mid-adolescence for training and cortical thickness at early adulthood for testing, from the same participants) was to identify those areas in which spatially discriminating features between the two groups at mid-adolescence could be used to predict subsequent between-group spatially discriminating features (at early adulthood). Results (Fig. 3) show brain areas in which patterns of spatially discriminating features best distinguished between the two groups, i.e., those with the highest SVM weights (‘top 5%’, 4009 vertices).

To assess the extent of overfitting due to within-subject correlation, we trained and tested an additional SVM classifier using participants who were included in either training or testing phase only. The training phase used cortical thickness from randomly selected 42 controls and 42 preterm individuals scanned at mid-adolescence, and the testing phase involved cortical thickness from newly and randomly selected 42 controls and 42 preterm-born individuals scanned at early adulthood. Classification accuracy was calculated using leave-one-out cross validation. This was repeated 1000 times (randomly selecting the control and preterm-born individuals on each permutation), calculating classification accuracy at each permutation. Then, mean classification accuracy across all permutations was compared with classification accuracy of the original SVM described above. Additionally, the 5% of vertices that contributed most to classification on each permutation were saved and the spatial overlap between these vertices and the top 5% of vertices obtained using the original SVM classification was calculated for each permutation. The average spatial overlap percentage between the top 5% of vertices from the original SVM and each permuted training/test data set was computed to assess the robustness of results and to test generalizability of predictive regions.

#### Longitudinal changes in cortical thickness and neuropsychological outcome

For every participant, and in each hemisphere, cortical thickness change was calculated by performing a vertex-wise subtraction of cortical thickness measured at mid-adolescence from cortical thickness measured at early adulthood. This involved only the participants who were scanned at both time points (i.e., those participants used in the SVM analysis).

Subsequently, an inclusive mask of 1% of the brain with the highest SVM weights was created (‘top 1%’, 803 vertices), in order to confine the structure-function analyses to those areas displaying the most pronounced between-group differences in patterns of spatially discriminating features. These areas were regarded as the most promising possible biomarkers of adult cognitive outcome. Within the whole ‘top 1%’ mask, vertex-wise Pearson correlations were performed between cortical thickness change and the cognitive scores that differed statistically between the groups at early adulthood, in control and preterm participants separately. Full-scale IQ was significantly associated with global EF scores (r = 0.47, p < 0.0001), hence it was not independently investigated in the subsequent analyses. In Results we report clusters containing ≥ 20 vertices in which cortical thickness change significantly correlated with our chosen outcome measure.

## Results

### Participants' neonatal, socio-demographic and neuropsychological outcome data

Table 1 shows participants' neonatal and socio-demographic details. The two groups did not differ statistically in sex and socio-economic status, but they differed in age, with preterm-born participants being slightly older than controls. Preterm-born participants who were assessed only at mid-adolescence did not differ from those assessed at both time points in gestational age (F_(1, 158)_ = 2.70, p = 0.10) and birth weight (F_(1, 158)_ = 1.78, p = 0.19), as revealed by univariate analysis of variance.

Controls performed significantly better than VPT individuals in all neuropsychological subtests, except the Recall lists A and B of the CVLT (Table 2). Global EF and global memory scores were also significantly higher in controls.

When only those participants who were scanned at both time points were compared, controls scored significantly higher than VPT individuals in IQ and COWAT, as well as in global EF.

### Between-group cross-sectional cortical thickness analyses

Between-group cross-sectional analyses showed that at mid-adolescence, the VPT group had significantly reduced cortical thickness compared to controls in bilateral parahippocampal regions and left insula (Fig. 1a). At early adulthood, cortical thickness was still significantly reduced in these regions in the VPT group, with the addition of areas centred in right temporo-parietal junction and the posterior part of the right inferior frontal sulcus (Fig. 1c).

Cortical thickness was significantly greater in widespread areas in the VPT group at mid-adolescence, and especially in prefrontal areas, occipital and temporal cortices and insula (Fig. 1b). At early adulthood, only cortical thickness in left temporal pole and a smaller right ventromedial prefrontal cortical area (centred on the medial orbitofrontal cortex) were significantly greater in the VPT group (Fig. 1d). All the areas displaying significant between-group differences in mean cortical thickness at mid-adolescence and early adulthood are shown in Table 3. All between group differences show medium to large effect sizes.

### Within- and between-group longitudinal cortical thickness analyses

Within-group longitudinal cortical thickness analyses showed that, in both groups, cortical thickness significantly decreased from mid-adolescence to early adulthood, after controlling for sex. In controls, cortical thickness decrease occurred predominantly in posterior (bilateral) and medial (left) aspects of frontal cortex, parietal areas and ventral aspects of temporal lobes bilaterally (Fig. 2a). In the VPT group, a more pronounced decrease occurred in similar, but much more widespread regions (Fig. 2b).

Between-group longitudinal cortical thickness analyses using a group-by-time point interaction design (univariate approach), adjusting for sex, were non-significant. That is, overall cortical thickness change from mid-adolescence to early adulthood did not significantly differ between controls and preterm born individuals at any individual cortical vertex.

### Longitudinal prediction of cortical thickness alterations using SVM

Results of SVM analyses (multivariate approach) demonstrated 86.5% mean classification accuracy. The ‘top 5%’ regions (i.e., 5% of vertices with the highest SVM weights) which spatially discriminated between the groups included predominantly bilateral temporal poles, inferior temporal gyri, superior parietal lobes, right occipito-temporal and lingual gyri, as well as left medial frontal areas (Fig. 3). In most of these areas, VPT individuals displayed greater relative decrease (or smaller increase) in cortical thickness than controls, except mainly around bilateral temporal poles, where cortical thickness decrease was smaller in the VPT group.

To assess the extent of potential overfitting due to within-subject correlation, which accompanies longitudinal measures, 1000 additional SVM analyses were performed involving 1000 different sets of VPT and control participants who were included in either the training or testing phase only. These additional SVM analyses had a mean classification accuracy of 86.3% (SD = 3.6%). There was no significant difference in classification accuracy between the original SVM (using training data at mid-adolescence and testing data from the same participants at early adulthood) and the additional SVM analyses using training and testing data from different sets of participants (confidence level: 95%). There was an average 95.3% (SD = 8.9%) overlap between the ‘top 5%’ regions of the additional SVM across all permutations and the ‘top 5%’ regions obtained by the original SVM.

### Longitudinal cortical thickness change and cognitive scores at early adulthood

Results of the vertex-wise correlations carried out in each group between cortical thickness change in the ‘top 1%’ regions and global EF scores at early adulthood identified 3 significant clusters (< 20 vertices).

**Fig. 4:**
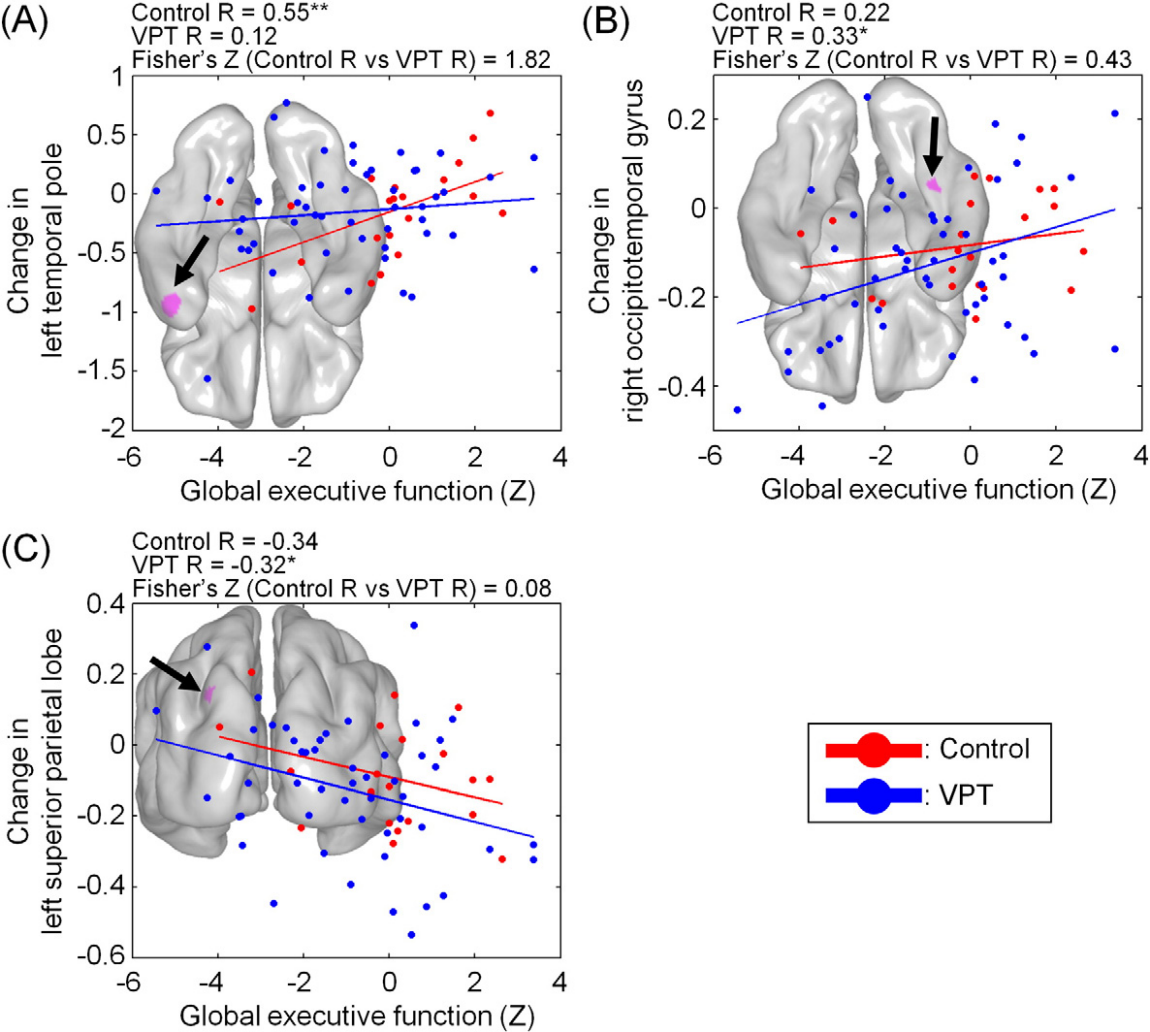
Correlation between cortical thickness change (in millimetres) and global executive function scores.

Controls' cortical thickness change in the left temporal pole (Fig. 4a) was significantly and positively associated with global EF score (r = 0.55, p = 0.009), i.e. cortical thinning of the temporal pole was associated with lower EF scores. The preterm group displayed a non-significant positive correlation. The correlations did not significantly differ between groups, although the p value was at borderline significance levels (Fisher's Z = 1.82, p = 0.068). Adjusting for baseline cortical thickness in left temporal pole did not alter these associations (controls, r = 0.60, p = 0.006; VPT, r = 0.10, p = 0.48).

The VPT group showed a significant positive correlation between cortical thickness change in the right occipitotemporal gyrus and global EF scores (r = 0.33, p = 0.018), i.e. cortical thinning of the right occipitotemporal gyrus was associated with lower EF scores (Fig. 4b). In the left superior parietal lobe, a negative correlation was found (r = − 0.32, p = 0.020) (Fig. 4c). In both regions, the control group showed correlations which were non-significant and in the same direction as the preterm group. Controlling for baseline cortical thickness region-specific values did not alter these associations: the VPT group showed a significant positive correlation between global EF score and CT change in right occipitotemporal gyrus (r = 0.30, p = 0.03), whilst results were not statistically significant for controls (r = 0.27, p = 0.24); cortical thickness change in left superior parietal lobe was negatively correlated with global EF score in VPT participants (r = − 0.28, p = 0.04); results were non-significant for controls (r = − 0.32, p = 0.17).

None of the other cognitive scores which significantly differed between very preterm-born participants and controls at early adulthood were significantly associated with cortical thickness change in the ‘top 1%’ regions identified by SVM.

### Longitudinal cortical thickness change and gestational age

Results of vertex-wise correlations carried out in the VPT group between cortical thickness change in the ‘top 1%’ regions and gestational age identified only one significant cluster. This was centred in the left superior parietal lobe, where more pronounced cortical thinning from mid-adolescence to early adulthood was correlated with older gestational age (r = − 0.33; p = 0.02).

## Discussion

In this investigation we extend the findings of cross-sectional studies on cortical thickness following VPT birth by demonstrating for the first time, with a longitudinal design, that patterns of cortical development differ between VPT individuals and controls from mid-adolescence to early adulthood, using well established analytical methods. We further show that cortical developmental alterations have important functional implications, as they are associated with scores on executive function tests.

### Cross-sectional and longitudinal cortical thickness measurements

Results of cross-sectional analysis revealed both increases and decreases in cortical thickness in VPT individuals compared to controls. VPT adolescents had greater cortical thickness compared to controls in several brain regions, including large sections of the frontal cortex, occipital, temporal and insular cortices. By early adulthood, the number and magnitude of areas showing greater cortical thickness in the VPT group substantially decreased, although areas of increased cortical thickness were still present in left temporal pole and right medial orbitofrontal cortex. These results suggest a delayed cortical maturational trajectory in VPT individuals, as has been observed in other neurodevelopmental conditions, such as attention deficit hyperactivity disorder (ADHD) (Shaw et al., 2007). The hypothesis of delayed cortical maturation is supported by our longitudinal findings, which showed that although in both groups cortical thickness decreased from mid-adolescence to the beginning of early adulthood, following developmental patterns observed in normative samples (Shaw et al., 2008; Tamnes et al., 2010), these changes were much more extensive in VPT individuals, resulting in fewer areas demonstrating significant between group differences at early adulthood. These results are consistent with findings of other studies investigating cortical thickness in similar samples (Martinussen et al., 2005; Bjuland et al., 2013) and with our previous findings of decreased cerebellar volume and surface area of the corpus callosum in the same VPT individuals studied here compared to controls at mid-adolescence, but not at early adulthood (Allin et al., 2007; Parker et al., 2008).

However, results also showed thinner cortex in large portions of bilateral medial temporal regions, including the parahippocampal, entorhinal and perirhinal cortices in right hemisphere, and insula in left hemisphere at both time points in the VPT group compared to controls. These results indicate that selective cortical and subcortical areas could be particularly vulnerable to alterations persisting into early adulthood in VPT/VLBW samples (Skranes et al., 2012; Bjuland et al., 2013; Nosarti et al., 2014).

The mechanisms underlying cortical alterations in the VPT group remain unclear, but could reflect altered neuronal differentiation in the cortical plate, a transient structure which is present in the third trimester of gestation, during which extensive afferent fibers migrate from it into the cortex to form their final connections, and play a fundamental role in the establishment of early neuronal networks (Kostovic et al., 2011). Evidence from both animal and human research in fact suggests that the cortical plate may be vulnerable to pre- and perinatal events (Miller and Ferriero, 2009). Alternatively, cortical alterations may be secondary to white matter injury, which has been associated with cortical reduction and neuronal loss (Andiman et al., 2010). Environmental disturbances of such critical developmental processes may have subsequent life-long consequences for cortical development, as well as underlie an elevated risk for cognitive deficits and psychopathology (i.e., represent a ‘pre-symptomatic signature’) (Ben-Ari, 2008).

In terms of regional specificity, cross-sectional results demonstrated cortical alterations in the VPT group predominantly in temporal cortex, insula and frontal regions. These results may be interpreted with reference to differential timing of human prenatal cortical formation (Miller et al., 2014) and the observation that temporal and frontal cortices appear to be particularly susceptible even to subtle environmental perturbations in utero (Raznahan et al., 2012). Furthermore, frontal and temporal cortices show protracted maturational changes in comparison to other brain regions (Shaw et al., 2008; Petanjek et al., 2011), and this protracted development considerably lengthens the period of neural vulnerability, as complex dynamic molecular and cellular events increase the risk of damage to numerous targets (Petanjek and Kostovic, 2012).

In terms of functional correlates, frontal and temporal cortices belong to heteromodal areas of the brain, which are made up by reciprocally interconnected regions responsible for integrating sensory information into high-order cognitive processes (Mesulam, 1998). Cortical maturational delays in heteromodal cortices may be partly responsible for the cognitive deficits observed in VPT-born samples (Nosarti et al., 2007; Bless et al., 2013; Heinonen et al., 2013), as cortical thinning is thought to reflect selective removal of synapses that may contribute to the establishment of neural circuits supporting cognitive processes (Hensch, 2004; Knudsen, 2004; Shaw et al., 2007; Raznahan et al., 2011). Specifically, cortical alterations in prefrontal regions may underlie the increased prevalence of deficits in high order ‘executive’ functions affecting information processing, attentional control, cognitive flexibility and goal setting that have been described in preterm samples (Nosarti et al., 2008; Burnett et al., 2013; White et al., 2014). Cortical alterations in temporal regions may further underlie verbal and non-verbal memory impairments reported in early adulthood in preterm born individuals (Nosarti et al., 2014; Aanes et al., 2015). Similarly, cortical maturational delays in insula, which has been described as critical for emotional awareness (Gu et al., 2013) may explain poor social competence described in VPT adolescents (Healy et al., 2013).

In addition to their central involvement in high-order cognitive functions, alterations in fronto-temporal networks have been associated with an increased vulnerability to develop psychiatric problems in a variety of clinical and sub-clinical samples, including individuals with schizophrenia, those at risk of psychosis (Borgwardt et al., 2007; Benetti et al., 2009) and ADHD (Shaw et al., 2007). Reduction in grey matter in temporal cortices has further been associated with early-life psychosocial adversities in individuals without a current psychiatric diagnosis (Walsh et al., 2014). As the altered patterns of cortical development following VPT birth observed here implicate similar brain cortices to those described in psychiatric disorders known to be more prevalent in VPT samples than controls (Nosarti et al., 2012; Johnson and Wolke, 2013), we speculate that an increased risk of developing psychiatric disorder following VPT birth may be underpinned by region-specific altered cortical development. Support to the intermediate phenotype hypothesis has been provided by other studies including one by Shaw et al. (2011) which observed neurodevelopmental changes in cortical thickness resembling those found in ADHD in typically developing youth exhibiting hyperactive/impulsive signs (Shaw et al., 2011).

### Prediction of cortical thickness alterations by machine learning

Despite seemingly apparent differences in longitudinal cortical thickness changes between VPT individuals and controls, conventional mass-univariate statistical methods did not reveal any statistically significant between-group differences. This could be due to the fact that these methods possess relatively high exploratory power, as they focus on changes throughout the brain, but provide modest statistical power by applying stringent multiple comparisons corrections to avoid Type I errors (Ecker et al., 2010). We therefore used a multivariate approach, SVM, in order to identify significant spatially discriminating features between the two groups, which yielded a classification accuracy of 86.5%. A further advantage of SVM compared to mass-univariate statistical methods is that it allows us to make inferences at the individual, rather than the group level. The regions highlighted by SVM analysis (top 5%) included temporal, occipital and frontal cortices. Within these regions, we further selected the ‘top 1%’, regarding these as the most vulnerable to long-term alterations following VPT birth. Cortical thickness changes in the ‘top 1%’ were significantly associated with scores on EF tests at early adulthood, which were significantly lower in VPT individuals compared to controls, in line with previous studies (Nosarti et al., 2008; Burnett et al., 2013; White et al., 2014). Therefore, as we used a predictive analysis approach, our results identified a potential biomarker of important changes in cortical development influencing cognitive outcomes.

Results showed differential associations between region-specific cortical thickness longitudinal changes and EF scores in the VPT and control groups. Overall, cortical thickness changes resembling developmental trajectories in control samples correlated with higher EF scores, consistent with our previous findings demonstrating that every 25% difference in regional volume in VPT adolescents compared to controls was associated with a significant increased risk of cognitive impairment (Nosarti et al., 2008). However, both EF tests used in the current study were language based and assessed specific aspects of EF only, namely response inhibition (HSCT), mental flexibility and the ability to use different cognitive strategies, such as clustering (Spreen and Strauss, 1991) (COWAT). Therefore, our current results refer to specific EF subcategories only and may show specific correlation patterns not true for other tests tapping into other aspects of EF that were not studied here.

Furthermore, all the areas where significant associations between cortical thickness change and EF scores were observed, form part of networks underpinning the cognitive and behavioural sequelae of VPT birth (Cheong et al., 2013; Simms et al., 2013; Wilson-Ching et al., 2013; Fischi-Gomez et al., 2014): the temporal pole is part of a cortico-striatal-thalamo-cortico network (Fan et al., 2013) involved in social processing, multimodal sensory integration, and high-order executive functions (Elliott, 2003; Mills et al., 2014); the occipitotemporal cortex is implicated in skilled reading and selective attention (Shaywitz et al., 2004; Nelissen et al., 2013), and the superior parietal cortex is associated with numerical information processing, executive functions and sentence comprehension (assessed here with the HSCT) (Meyler et al., 2008; Otsuka et al., 2008; Rosenberg-Lee et al., 2014). The temporal pole, occipitotemporal cortex and superior parietal cortex have further been implicated in different aspects of language (Ardila et al., 2015; Brownsett and Wise, 2010), therefore the significant association between regional cortical changes and EF scores seen here may be due to the type of EF tests we used, which were language-based. Alternatively, significant associations between cortical changes and outcome, in areas that are not typically involved in executive functions, could be interpreted in the context of an altered development of the entire brain following VPT birth, which could be characterized by “different functional structures” (Thomas and Karmiloff-Smith, 2003).

Results of cross-sectional studies have also reported significant associations between smaller cortical thickness in parahippocampal region and lower IQ in VPT/VLBW adolescents (Martinussen et al., 2005) and smaller cortical thickness in entorhinal cortex in VPT/VLBW adults and low EF scores (Skranes et al., 2012).

Our analyses further revealed that individuals with the youngest gestational age displayed less parietal cortical thinning in left superior parietal cortex compared to those born nearer term, suggesting region-specific developmental delays associated with increased neonatal risk. A recent cross-sectional study reported a significant positive association between length of gestation and increased local network efficiency, particularly in bilateral superior parietal cortex (and precuneus) (Kim et al., 2014), which was interpreted by the authors as underlying the impact of length of gestation on structural hubs that are critical for global neural communication and integration (Collin et al., 2014).

Participants included in this study were born between 1979 and 1984; since then substantial changes in neonatal care have occurred (e.g., use of exogenous surfactants, antenatal steroids, mechanical ventilation) (Flor-de-Lima et al., 2012). Therefore, the VPT individuals studied here may not be representative of contemporary cohorts, who may have improved neurodevelopmental outcomes (Serenius et al., 2013). However, studies with younger samples have reported alterations in similar cortical areas (Murner-Lavanchy et al., 2014), supporting the idea of regional vulnerability following VPT birth. A further limitation is the inclusion of term-born controls from the general population, which could differ from our study group in variables that have not been measured.

## Summary and conclusion

The findings of this study show that cortical development from mid-adolescence to early adulthood is altered in individuals who were born VPT. Vulnerability of cortical development shows regional specificity, affecting predominantly frontal, temporal cortices and insula. Nevertheless, as cortical development continues beyond adolescence (Gogtay et al., 2004; Kochunov et al., 2011; Lebel and Beaulieu, 2011; Petanjek et al., 2011), it remains to be ascertained whether these alterations reflect neurodevelopmental delays or long lasting structural alterations associated with VPT birth.

The findings of this study further suggest that longitudinal alterations in cortical thickness are associated with cognitive outcome. The study of altered cortical developmental trajectories could be therefore useful in predicting which cognitive functions may be better candidates for focussed training in VPT samples (Pascoe et al., 2013), which could target the neuroplastic capacity of late maturing cortices involved in specific high order cognitive functions (Petanjek et al., 2011). Mapping dynamic cortical changes throughout critical phases of development following VPT birth could further aid to the identification of individuals at high-risk for cognitive impairment, who could be prospectively identified and closely monitored — to decide if, when and what interventions may be appropriate.

## Figures and Tables

**Table 1:** Participants' neonatal and socio-demographic details.

Number of participants		All assessed at mid-adolescence			All assessed at early adulthood				Assessed at both time points		
		Control	Preterm	Statistics	Control	Preterm	Statistics		Control	Preterm	Statistics
		88	160		42	67			21	51	
Age (mean (SD))		15.0 (0.7)	15.2 (0.5)	F_(1, 246)_ = 7.74^a^	19.3 (1.2)	20.2 (1.2)	F_(1, 107)_ = 13.45^b^	*Time 1*	15.0 (0.7)	15.4 (0.5)	F_(1, 70)_ = 9.61^a^
								*Time 2*	19.2 (0.7)	20.1 (1.0)	F_(1, 70)_ = 14.44^b^
Male ratio (%)		55.7	51.9	*X*^2^_(1, N = 248)_ = 0.33	52.4	44. 8	*X*^2^_(1, N = 109)_ = 0.60		47.6	45.1	*X*^2^_(1, N = 72)_ = 0.04
Gestational age at birth (mean (SD))		40.2 (1.3)	29.1 (2.3)	F_(1, 222)_ = 1364.51^c^	40.2 (1.6)	28.8 (2.2)	F_(1, 99)_ = 705.94^c^		40.1 (1.7)	28.7 (2.3)	F_(1, 70)_ = 428.68^c^
Birth weight in grams (mean (SD))		3377 (428)	1285 (369)	F_(1, 215)_ = 1240.18^c^	3318 (391)	1214 (372)	F_(1, 97)_ = 671.54^c^		3287 (362)	1228 (388)	F_(1, 68)_ = 403.20^c^
Socio-economic status (number (%))	I–II	35 (39.8%)	59 (36.9%)	*X*^2^_(4, N = 248)_ = 1.08	22 (52.4%)	26 (38.8%)	*X*^2^_(4, N = 109)_ = 5.87	*Time 1*			*X*^2^_(4, N = 72)_ = 2.74
	III	24 (27.3%)	52 (32.5%)		12 (28.6%)	30 (44.8%)					
	IV–V	14 (15.9%)	27 (16.9%)		7 (16.7%)	9 (13.4%)		*Time 2*			*X*^2^_(3, N = 72)_ = 1.17
	Unclassified	8 (9.1%)	12 (7.5%)		0 (0.0%)	2 (3.0%)					
	Missing	7 (8.0%)	10 (6.3%)		1 (2.4%)	0 (0.0%)					

Univariate analysis variance was carried out to compare age, gestational age and birth weight between controls and preterm born individuals. For each contrast, F statistic value and degrees of freedom are reported. Distributions of gender and socio-economic status were also compared using Pearson Chi-Square test. Pearson Chi-Square value and degrees of freedom are reported.

a p < 0.01; ^b^ p < 0.001; ^c^ p < 0.0001.

**Table 2:** Neuropsychological outcome in very preterm-born individuals and controls.

	All assessed at early adulthood			Assessed at both time points		
	Mean (SD)		ANOVA	Mean (SD)		ANOVA
	Control (n = 42)	Preterm (n = 67)		Control (n = 21)	Preterm (n = 51)	
*Full scale IQ*						
WASI	105.5 (13.9)	96.4 (13.9)	F_(1, 107)_ = 11.05⁎	104.2 (14.3)	96.7 (14.5)	F_(1, 70)_ = 4.00⁎
*Global memory*^a^		*− 2.6 (5.1)*	*F_(1, 107)_ = 8.31*⁎		*-1.9 (5.4)*	*F(1, 70) = 2.13*
CVLT (Recall (list A))	56.3 (8.8)	53.5 (9.8)	F_(1, 107)_ = 2.25	54.9 (9.0)	54.7 (10.1)	F_(1, 70)_ = 0.01
CVLT (Recall (list B))	6.5 (1.9)	6.2 (2.1)	F_(1, 107)_ = 0.77	6.4 (2.2)	6.3 (2.2)	F_(1, 70)_ = 0.02
CVLT (Recognition hits)	15.2 (0.8)	14.7 (1.6)	F_(1, 107)_ = 4.01⁎	15.4 (0.7)	14.9 (1.5)	F_(1, 70)_ = 2.10
WMS (Immediate visual memory)	11.4 (2.1)	9.8 (3.3)	F_(1, 107)_ = 7.38⁎	11.0 (2.2)	9.8 (3.3)	F_(1, 70)_ = 2.38
WMS (Delayed visual memory)	10.6 (2.9)	8.5 (3.4)	F_(1, 107)_ = 10.57⁎	10.1 (2.9)	8.5 (3.4)	F_(1, 70)_ = 3.48
*Global executive function*^a^		*− 0.9 (1.8)*	*F_(1, 107)_ = 6.95*⁎		*− 1.0 (2.0)*	*F_(1, 70)_ = 4.15*⁎
HSCT (Scaled)	5.9 (1.5)	5.1 (2.0)	F_(1, 107)_ = 4.90⁎	5.7 (1.6)	4.9 (2.1)	F_(1, 70)_ = 2.05
COWAT	40.5 (10.6)	36.2 (9.7)	F_(1, 107)_ = 4.67⁎	40.9 (9.3)	35.8 (9.8)	F_(1, 70)_ = 4.10⁎

COWAT = Controlled Oral Word Association Test; CVLT = California Verbal Learning Test; HSCT = Hayling Sentence Completion Test; WASI = Wechsler Abbreviated Scale of Intelligence; WMS = Wechsler Memory Scale. Univariate analysis variance was carried out to compare scores between controls and preterm born individuals.

⁎ p < 0.05.

a Global scores are the sum of domain-specific Z scores; for VPT participants these were obtained using means and SDs from controls, which by default were set to 0 and 1. Only variables where VPT individuals showed significant differences from controls were used; ‘Global EF’: HSCT (Scaled) and COWAT scores; ‘Global memory’: CVLT (Recognition hits) and WMS (Immediate and Delayed) scores.

**Table 3:** Areas displaying significant between-group differences in mean cortical thickness (in millimetres) at mid-adolescence and early adulthood.

Mid-adolescence			
Region	VPT (n = 160), mean (95% CI)	Control (n = 88), mean (95% CI)	Cohen's d
Right parahippocampus	2.96 (2.91–3.02)	3.21 (3.14–3.28)	− 0.68
Left prahippocampus	2.55 (2.51–2.59)	2.76 (2.70–2.82)	− 0.80
Left insula	4.40 (4.35–4.46)	4.62 (4.53–4.70)	− 0.57
Left vmPFC/mOFC/Cingulate	3.55 (3.51–3.59)	3.37 (3.33–3.42)	0.75
Right temporal pole	3.86 (3.81–3.91)	3.63 (3.58–3.69)	0.76
Right vmPFC/dmPFC/mOFC	3.81 (3.76–3.85)	3.62 (3.57–3.67)	0.68
Left lingual/fusiform gyri	3.24 (3.20–3.27)	3.12 (3.08–3.15)	0.63
Left temporal pole	3.83 (3.77–3.89)	3.55 (3.48–3.61)	0.78
Left superior frontal gyrus	2.72 (2.68–2.77)	2.58 (2.53–2.63)	0.51
Left anterior insula	4.54 (4.46–4.63)	4.24 (4.15–4.34)	0.59
Right central sulcus	2.87 (2.84–2.91)	2.75 (2.70–2.81)	0.50
Left postcentral gyrus	2.43 (2.38–2.47)	2.29 (2.25–2.33)	0.54
Left central sulcus	2.81 (2.77–2.85)	2.68 (2.63–2.72)	0.50
Right middle frontal gyrus	2.93 (2.88–2.98)	2.79 (2.74–2.85)	0.46
Right occipito-temporal sulcus	3.59 (3.54–3.64)	3.46 (3.41–3.51)	0.47

**Early adulthood**			

**Region**	**VPT (n = 67), mean (95% CI)**	**Control (n = 42), mean (95% CI)**	**Cohen's d**

Right parahippocampus	2.99 (2.90–3.08)	3.32 (3.22–3.42)	− 0.94
Left parahippocampus	2.89 (2.81–2.97)	3.21 (3.09–3.33)	− 0.89
Right temporo-parietal junction	3.2 (3.15–3.25)	3.41 (3.35–3.47)	− 1.04
Left insula	3.88 (3.80–3.97)	4.16 (4.09–4.22)	− 0.91
Right inferior frontal sulcus	3.09 (3.04–3.14)	3.32 (3.26–3.38)	− 1.09
Left temporal pole	3.71 (3.63–3.80)	3.39 (3.29–3.49)	0.95
Right medial orbitofrontal cortex	2.82 (2.73–2.90)	2.57 (2.48–2.66)	0.77

vmPFC/mOFC = ventromedial prefrontal cotex; mOFC = medial orbitofrontal cortex; dmPFC = dorsomedial prefrontal cotex.
